# Gender difference in the association between cough severity and quality of life among patients with postinfectious cough

**DOI:** 10.1186/s12955-021-01680-5

**Published:** 2021-01-26

**Authors:** Wei Liu, Qinqin Wu, Bing Mao, Hongli Jiang

**Affiliations:** 1 Pneumology Group, Department of Integrated Traditional Chinese and Western Medicine, West China Hospital, Sichuan University, No.37 Guoxue Street, Chengdu, 610041 China; 2 Health Management Center, West China Hospital, Sichuan University, No.37 Guoxue Street, Chengdu, 610041 China; 3 Department of Pulmonary Diseases, State Key Laboratory of Biotherapy of China, West China Hospital, Sichuan University, 1 Keyuansi Road, Chengdu, 610041 China

**Keywords:** Postinfectious cough, Cough specific quality of life, Cough severity, Gender difference

## Abstract

**Background:**

Despite close link exists between cough severity and quality of life (QoL), whether gender difference is implied in the effect of cough on QoL has not been studied yet. This study primarily aims to investigate whether the association between cough severity and QoL is modified by gender in patients with postinfectious cough.

**Methods:**

Secondary analyses were performed in 180 participants with postinfectious cough in a multisite randomized controlled trial. Baseline demographics, clinical characteristics and score of cough specific quality of life questionnaire (CQLQ) were collected. Linear regression analyses were conducted to examine gender difference in CQLQ score and the association between cough severity and CQLQ score.

**Results:**

Difference between women and men was not significant in CQLQ total score in the unadjusted analysis (*P* = 0.077). Women had a 2.20-point higher CQLQ total score than men (95% confidence interval (CI) 0.11–4.30; *P* = 0.039), after adjusting for age, cough duration, cough severity, and clinical center. Gender significantly modified the association between cough severity and CQLQ total score (coefficient 1.80, 95% CI 0.29–3.30; *P* = 0.020), after adjusting for age, cough duration, and study center. An increase of 1-point in cough severity was associated with a 2.55-point (95% CI 1.16–3.95) increase in CQLQ total score in women versus a 1.26-point (95% CI 0.20–2.31) increase in men (*P* = 0.020).

**Conclusions:**

Female sex may be associated with worse QoL than men, and women’s QoL may be more significantly impaired as cough symptom deteriorates. Gender difference should be taken into account in the clinical settings and research of cough and cough related QoL.

*Trial registration*: Chinese Clinical Trial Registry, ChiCTRTRC12002297. Registered 19 June 2012, http://www.chictr.org.cn/abouten.aspx.

## Background

Cough is among the most common respiratory symptoms that lead patients to seek medical help in primary care and respiratory specialist clinics [1–4]. Although fatal complications are rare, unremitting cough is always frustrating and bothersome. Loss of sleep, exhaustion, irritability, urinary incontinence, cough syncope, social disability, and inability to perform daily activities are some of the negative outcomes associated with considerable distress in patient's daily life and significant impairments in health-related quality of life (QoL) [5, 6]. With quantitative or semi-quantitative methods, the changes in QoL in cough populations can be accurately analyzed. Commonly used validated instruments include the Cough Specific Quality of Life Questionnaire (CQLQ) [7], the Leicester Cough Questionnaire [8], and the Chronic Cough Impact Questionnaire [9]. These questionnaires have multiple items and domains and address the impact of cough on various aspects of health-related QoL, including physical, psychological, and social domains [10]. As such, QoL is closely affected by cough severity [11].

Most studies show a higher prevalence of nocturnal and non-productive cough in women than in men [12, 13]. Besides, previous studies have showed that physiology, physiopathology and outcome measures of cough differ in many ways between males and females [14–20]. These could originate from gender-related differences not only in anatomy and physiology of respiratory, immune or nervous system, but also in behavioral and socio-cultural factors that considerably affect respiratory health [21]. However, whether the impact of cough on QoL is also variable between genders is still controversial.

Patients who complain a persistent cough lasting > 3 weeks after experiencing the acute symptoms of an upper respiratory tract infection may have a postinfectious cough. It is the most common cause of subacute cough, accounting for 30–50% of patients with subacute symptoms [22, 23]. We recently demonstrated that QingfengGanke granule improved clinical symptoms and cough specific QoL over placebo in patients with postinfectious cough [24]. Although a worse QoL has been reported in women with chronic cough than men [17], a similar cough related QoL between gender is demonstrated during acute conditions such as upper respiratory infections [25]. The gender difference in cough related QoL in subacute cough patients has not been studied yet.

In this post-hoc analysis, we sought to investigate that whether health related QoL was different among women and men, and whether female sex was a modifier in the association between cough severity and QoL among patients with postinfectious cough.

## Methods

### Study sample

The original trial was a multisite randomized controlled trial (Chinese Clinical Trial Registry: ChiCTRTRC12002297). Detailed study methods had been previously reported [11]. Briefly, a total of 180 eligible participants were randomly assigned to three groups to assess efficacy and safety of a Chinese herbal prescription in patients with postinfectious cough. All patients provided written informed consent and were recruited from five tertiary hospitals across China. The original trial was approved by the Medical Ethics Committee of West China Hospital of Sichuan University (Chengdu, China). Patient enrollment started in April 2011 and ended in March 2012. The current analysis included baseline data of all 180 participants.

### Variables

Demographic variables including gender, age, and marital status were collected; body mass index was calculated based on the reported height and weight; clinical characteristics including previous physician-diagnosis of postinfectious cough, medication use for previous postinfectious cough attack, and cough duration were recorded; cough severity was measured by visual analogue scale (VAS), a widely used tool in clinical research and practice; cough symptom score assessed cough frequency, severity and the overall impact, and was evaluated in terms of daytime and nighttime intervals [26]; health related QoL was assessed using CQLQ at baseline. The questionnaire contains 28 items, each item being scored on a 4-point scale. All these items are summarized into six subscales: physical complaints, psychosocial issues, functional abilities, emotional well-being, extreme physical complaints, and personal safety fears. The score ranges from 28 and 112, with lower scores indicating a better QoL [7].

### Statistical analysis

Baseline results were presented as mean (standard deviation) or median (interquartile range) for continuous variables as appropriate, and frequency (percentage) for categorical variables. Characteristics of women and men were compared using two-sample *t* test or Mann–Whitney U test for continuous variables and chi-square test for categorical variables.

Cough VAS and cough duration were included in the multiple linear regression model, based on their known or potential impact on QoL [25, 27, 28]. Clinical center was additionally adjusted for possible clustering effects. As current smokers and recent ex-smokers were excluded from the trial, smoke status was not included as a covariate. Daytime and nighttime cough symptom scores were also not included in the model because they captured cough severity and overall impact and might highly correlated with cough VAS. The interactions between gender and cough VAS in the association of total CQLQ were analyzed in the above-mentioned models. The multivariate linear regression analyses were repeated to assess the interaction between gender and cough VAS in the association of six subscales. Regression diagnostics were performed to examine the fit, multicollinearity and potentially influential observations.

A two-tailed *P* value < 0.05 was considered statistically significant. All analyses were performed using IBM SPSS Statistics version 21.0 (IBM, Armonk, NY).

## Results

### Demographic and clinical characteristics

A total of 104 women and 76 men were included in this study. There was no significant gender difference in age, body mass index and marital status. Women had longer cough duration than men did (32.6 days vs. 29.3 days, *P* = 0.035). Previous postinfectious cough history, medication use for previous cough attack, daytime and nighttime cough symptom score, cough VAS and CQLQ total score were not significantly different between women and men. The detailed demographics and clinical characteristics between women and men were shown in Table 1.

**Table 1:** Characteristics of women and men with postinfectious cough

Variables	Overall (*n* = 180)	Women (*n* = 104)	Men (*n* = 76)	*P* value
Age (years), mean ± SD	44.3 ± 12.6	45.0 ± 12.1	43.3 ± 13.2	0.385
Body mass index (kg/m^2^), mean ± SD	23.4 ± 3.1	23.1 ± 3.0	23.8 ± 3.2	0.131
Married, n (%)	157 (87.2)	91 (87.5)	66 (86.8)	0.896
Medication use for previous PIC, n (%)	27 (15)	20 (19.2)	7 (9.2)	0.063
Ever previously diagnosed with PIC, n (%)	29 (16.1)	17 (16.3)	12 (15.8)	0.920
Cough duration (days), mean ± SD	31.2 ± 9.0	32.6 ± 9.7	29.3 ± 7.5	0.035
Cough symptom score, median (IQR)				
Daytime	2 (2, 2)	2 (2, 2)	2 (2, 2)	0.379
Nighttime	2 (1, 2)	2 (1, 2)	2 (1, 2)	0.220
Cough VAS, median (IQR)	6 (5, 7)	6 (5, 7)	6 (5, 7)	0.060
CQLQ, mean ± SD				
Total	64.5 ± 7.9	65.4 ± 7.4	63.3 ± 8.3	0.077
Physical complaints	21.5 ± 3.0	21.8 ± 2.8	21.1 ± 3.2	0.160
Psychosocial issues	12.8 ± 2.1	12.9 ± 2.0	12.8 ± 2.3	0.723
Functional abilities	11.1 ± 2.0	11.2 ± 1.9	11.0 ± 2.2	0.527
Emotional well-being	7.2 ± 1.6	7.3 ± 1.6	7.0 ± 1.7	0.218
Extreme physical complaints	8.6 ± 1.5	9.0 ± 1.5	8.1 ± 1.3	< 0.001
Personal safety fears	6.9 ± 1.7	7.0 ± 1.7	6.8 ± 1.7	0.562

*CQLQ* cough specific QoL questionnaire, *IQR* interquartile range, *PIC* postinfectious cough, *VAS* visual analogue scale

### Gender difference in QoL

A higher mean score of extreme physical complaints was reported in women (9.0 vs. 8.1, *P* < 0.001). The difference between women and men was not significant in the score of total CQLQ and other five subscales in unadjusted analyses (Table 1). Women had a mean of 2.20-point higher CQLQ total score than men (95% confidence interval (CI) 0.11–4.30; *P* = 0.039), after adjusting for age, cough duration, cough VAS, and clinical center (R^2^ = 0.297). Female sex was significantly associated with a 0.88-point increase in extreme physical complaints score compared to men in the adjusted analysis (95% CI 0.47–1.28; *P* < 0.001). No significant difference between women and men was found in the other subscales analyses. Detailed differences in women versus men in the score of total CQLQ and six subscales in unadjusted and adjusted analyses were shown in Table 2.

**Table 2:** Gender differences in QoL in unadjusted and adjusted analyses

Variables	Unadjusted			Adjusted		
	Coefficient	95% CI		Coefficient	95% CI	
Total CQLQ	2.10	− 0.23	4.42	2.20^*^	0.11	4.30
Physical complaints	0.63	− 0.25	1.51	0.63	− 0.13	1.38
Functional abilities	0.19	− 0.41	0.80	0.36	− 0.22	0.95
Psychosocial issues	0.11	− 0.51	0.73	0.24	− 0.34	0.81
Emotional well-being	0.31	− 0.18	0.80	0.38	− 0.11	0.88
Extreme physical complaints	0.91^*^	0.50	1.33	0.88*	0.47	1.28
Personal safety fears	0.15	− 0.36	0.65	− 0.09	− 0.54	0.37

*P < 0.05

### Gender difference in the association between cough severity and QoL

**Fig. 1:**
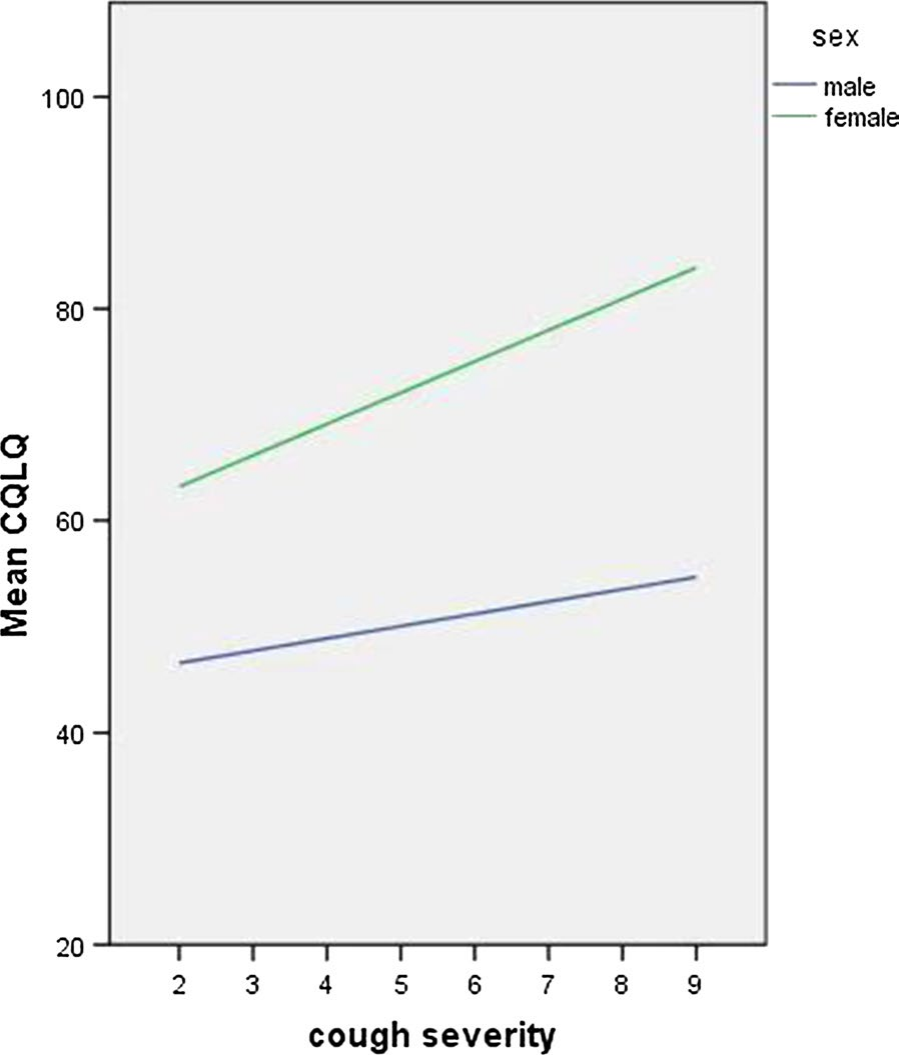
Association between cough severity and mean CQLQ total score between different genders

The association between cough VAS and CQLQ total score was marginally significantly different between women and men in unadjusted analysis (coefficient 1.67, 95% CI − 0.02–3.36; *P* = 0.053). After adjusting for age, cough duration, and study center, gender significantly modified the association between cough VAS and CQLQ total score (coefficient 1.80, 95% CI 0.29–3.30; *P* = 0.020) (Table 3). An increase of 1-point in cough severity assessed by VAS was associated with a 2.55-point (95% CI 1.16–3.95) increase in CQLQ total score in women versus a 1.26-point (95% CI 0.20–2.31) increase in men (*P* = 0.020). Women had a higher mean CQLQ total score than men with a similar degree of cough severity (Fig. 1).

**Table 3:** Gender differences in association between VAS and QoL

Variables	Women	Men	Interaction	*P* value
Total CQLQ				
Unadjusted	2.40 (1.04, 3.76)	0.73 (− 0.32, 1.78)	1.67 (− 0.02, 3.36)	0.053
Adjusted	2.55 (1.16, 3.95)	1.26 (0.20, 2.31)	1.80 (0.29, 3.30)	0.020
Physical complaints				
Unadjusted	0.99 (0.48, 1.50)	0.32 (− 0.07, 0.71)	0.67 (0.04, 1.30)	0.038
Adjusted	1.12 (0.58, 1.66)	0.58 (0.21, 0.94)	0.76 (0.21, 1.30)	0.006
Functional abilities				
Unadjusted	0.59 (0.23, 0.96)	0.37 (0.11, 0.63)	0.23 (− 0.20, 0.66)	0.301
Adjusted	0.37 (− 0.04, 0.77)	0.34 (0.05, 0.63)	0.17 (− 0.26, 0.59)	0.445
Psychosocial issues				
Unadjusted	0.66 (0.29, 1.03)	0.18 (− 0.10, 0.46)	0.48 (0.03, 0.93)	0.037
Adjusted	0.73 (0.35, 1.11)	0.23 (− 0.06, 0.52)	0.42 (0.01, 0.84)	0.045
Emotional well-being				
Unadjusted	− 0.02 (− 0.32, 0.28)	0.02 (− 0.21, 0.25)	− 0.04 (− 0.41, 0.33)	0.839
Adjusted	− 0.12 (− 0.47, 0.23)	0.04 (− 0.21, 0.29)	− 0.00 (− 0.37, 0.36)	0.985
Extreme physical complaints				
Unadjusted	0.19 (− 0.04, 0.42)	0.03 (− 0.18, 0.24)	0.16 (− 0.16, 0.47)	0.322
Adjusted	0.24 (− 0.01, 0.50)	0.14 (− 0.08, 0.36)	0.20 (− 0.10, 0.50)	0.185
Personal safety fears				
Unadjusted	0.02 (− 0.28, 0.32)	− 0.23 (− 0.47, 0.01)	0.25 (− 0.12, 0.63)	0.185
Adjusted	0.18 (− 0.14, 0.50)	− 0.12 (− 0.35, 0.11)	0.36 (0.03, 0.69)	0.034

Gender difference was significant in the association between VAS and physical complaints (coefficient 0.67, 95% CI 0.04–1.30; *P* = 0.038), and psychosocial issues (coefficient 0.48, 95% CI 0.03–0.93; *P* = 0.037). After adjusting for age, cough duration, and study center, gender difference was significant in the associations between VAS and physical complaints (coefficient 0.76, 95% CI 0.21–1.30; *P* = 0.006), psychosocial issues (coefficient 0.42, 95% CI 0.01–0.84; *P* = 0.045), and personal safety fears (coefficient 0.36, 95% CI 0.03–0.69; *P* = 0.034) (Table 3).

## Discussion

To our knowledge, this study is the first attempt to examine gender difference in QoL and the association between cough VAS and QoL among patients with postinfectious cough. We found that female sex was associated with worse QoL than men, and female sex was a modifier in the association between cough severity and QoL among postinfectious cough patients.

The current study showed that an increase of 1-point cough VAS score was associated with a higher CQLQ total score in women than that in men, after adjusting for age, cough duration, and clinical center. To the best of our knowledge, this is the first study to report this association. The greater effect of cough on QoL in women may be due to higher scores in physical complaints, psychosocial issues, and personal safety fears. Coughing also causes other stressful and unpredictable occurrences, such as stress urinary incontinence, which is currently a social disease and predominantly affects women, leading to considerable physical and psychological morbidities [29, 30].

Although cough lasted longer in women than men, the change in CQLQ total score that was associated with one day increase in cough duration was not significantly different between women versus men in either unadjusted or adjusted analyses (data not shown). The lack of gender difference in the association between cough duration and CQLQ scores suggested that female gender might be not associated with worse QoL as cough lasted longer in postinfectious cough condition, which might be explained by the self-limiting property of postinfectious cough [31].

Inherent to the nature of the secondary analysis of existing data, our study has several limitations that warrant further discussions. First, cough frequency was not collected for the original analysis and therefore, was not included in the adjusting models. This unmeasured variable might confound the relationships addressed and, thus, result in a source of possible bias. Although the impact of objective cough frequency on health related QoL has been considered to be mild to moderate [32, 33], whether their association is different between women and men needs to be further investigated. Second, this study only included 180 patients among five sites in the same country. A larger sample size with more sites that distributed across different countries and regions would provide a more robust result allowing to uncover gender difference in the association between cough severity and QoL.

## Conclusions

Among patients with postinfectious cough, women may experience worse QoL than men, and their QoL may be more markedly affected by cough symptom. Clinicians should be aware of the greater impact of cough on QoL in women, therefore, gender difference should be taken into account in the study of cough and cough related QoL.

## Data Availability

Not applicable.

## Abbreviations

CI: Confidence interval
CQLQ: Cough specific quality of life questionnaire
QoL: Quality of life
VAS: Visual analogue scale
